# Editorial on the Article “Ishiguro A, Nomura O, Michihata N, Kobayashi T, Mori R, Nishiya K, Kaneko K, and Japan Pediatric Society Steering Committee of Board Examination. Research during Pediatric Residency Training: A Nationwide Study in Japan”

**DOI:** 10.31662/jmaj.2018-0062

**Published:** 2019-02-20

**Authors:** Apul Goel, Monica Agrawal

**Affiliations:** 1 Department of Urology, King George’s Medical University, Lucknow, India; 2 Department of Obstetrics and Gynecology, King George’s Medical University, Lucknow, India

**Keywords:** research, residency, medical education, publication, conference presentation

The authors need to be congratulated for evaluating scholarly activity among pediatric residents doing residency in Japan and for bringing this important issue for discussion ^(1)^. The authors evaluated the log books of all residents appearing for certification examination. This method of evaluation has provided an objective and truthful status.

What is unclear in this article is whether it is mandatory for residents in Japan to do some research projects during residency. In many countries, including India, it is mandatory for residents to do a research project that is submitted as a thesis. For many years, there has been a push to encourage the residents in India to do these projects in a better fashion and to submit their results in some PubMed-indexed journal as an original study ^(2)^. In spite of the best efforts, the output has been dismal ^(3)^. Even the 15.7% research paper publication output as reported by the authors seems fairly low. Moreover, this 15.7% publication data includes some articles that were published in non-indexed journals. If research output from all fields of science and engineering are combined, then Japan ranks among the top contributors ^(4)^.

Exposure and an opportunity for presentation are extremely important during residency. It is heartening that 90.7% residents presented in various conferences. Currently, with so many conferences happening and with residents getting ample chance to present papers, 90.7% results, as regards academic presentations in conferences, are expected.

The authors reported significant variation in research output depending on the type of hospital ^(1)^. The authors have rightly highlighted the importance of mentoring, and I totally agree with their observation ^(5)^.

An interesting observation made by the authors is that the research output may be influenced by the clinical workload on the residents ^(1)^. Although not elaborated, but it seems that residents with heavier clinical workloads get lesser time to devote to research activities. However, this may not be true. The far more busy government institutions publish more than the less busy private hospitals in India ^(3)^. One reason being that it is easy to generate studies with larger number of patients in busy hospitals.

Of late, there has been a lot of emphasis on paper writing and research. In fact, this push has been responsible for the rise of predatory journals and poor quality research ^(6)^. It is far more important to understand research than do it. Instead of giving disproportionate emphasis to paper writing, a more sensible approach would be to train residents in research methodology and interpreting data.

Although the presented data belong to pediatric residents in Japan, these results hold true for all residency programs irrespective of the subject (in this article, Pediatrics) and location (most countries).

## Article Information

### Conflicts of Interest

None
